# Resveratrol Relaxes Human Gastric Smooth Muscles Through High Conductance Calcium-Activated Potassium Channel in a Nitric Oxide-independent Manner

**DOI:** 10.3389/fphar.2022.823887

**Published:** 2022-01-25

**Authors:** Beata Modzelewska, Krzysztof Drygalski, Hady Razak Hady, Aleksandra Kiełczewska, Andrzej Chomentowski, Krzysztof Koryciński, Paulina Głuszyńska, Tomasz Kleszczewski

**Affiliations:** ^1^ Department of Biophysics, Faculty of Medicine, Medical University of Bialystok, Bialystok, Poland; ^2^ Clinical Research Center, Medical University of Bialystok, Bialystok, Poland; ^3^ Clinical Department of General and Endocrine Surgery, Medical University of Bialystok, Bialystok, Poland

**Keywords:** resveratrol, smooth muscles, gastric motility, potassium channels, nitric oxide

## Abstract

Resveratrol, as a polyphenolic compound that can be isolated from plants, and also a component of red wine has broad beneficial pharmacological properties. The aim was to investigate the role of nitric oxide and potassium channels in resveratrol-induced relaxation of human gastric smooth muscle. Gastric tissues were obtained from patients who underwent sleeve gastrectomy for severe obesity (*n* = 10 aged 21–48; BMI 48.21 ± 1.14). The mechanical activity from the muscle strips was detected under isometric conditions as the response to increasing concentrations of resveratrol before and after different pharmacological treatments. Resveratrol caused an observable, dose-dependent gastric muscle relaxation. The maximal response caused by the highest concentration of resveratrol was 83.49 ± 2.85% (*p* < 0.0001) of the control. Preincubation with L-NNA, L-NAME, or ODQ did not prevent the resveratrol-induced relaxation. Apamin, glibenclamide, 4AP or tamoxifen, did not inhibit the relaxing effect of resveratrol, as well. In turn, blocking BK_Ca_ by TEA, iberiotoxin, or charybdotoxin resulted in inhibition of resveratrol-induced relaxation (91.08 ± 2.07, *p* < 0.05; 95.60 ± 1.52, *p* < 0.01 and 89.58 ± 1.98, *p* < 0.05, respectively). This study provides the first observation that the relaxant effects of resveratrol in human gastric muscle strips occur directly through BK_Ca_ channels and independently of nitric oxide signaling pathways. Furthermore, there is considerable potential for further extensive clinical studies with resveratrol as an effective new drug or health supplement to treat gastrointestinal dyspepsia and other gastric hypermotility disorders.

## Introduction

Resveratrol, as a naturally occurring bioactive molecule, has become a popular subject of scientific interest due to its potential benefit functions. Resveratrol belongs to a large group of natural, plant-derived polyphenols and phytoestrogens and can be found e.g., in grapes, berries, or red wine (Berk et al., 2019; Repossi et al., 2020). Numerous studies report its: anti-proliferative, anti-oxidative, anti-inflammatory, cytoprotective, anti-microbial, anti-dyslipidemia, or anti-diabetic properties (Charytoniuk et al., 2018; Repossi et al., 2020). Moreover, due to multiple pharmacological effects and promising results of preclinical studies, in many clinical trials over the past few years, oral supplementation with combinations of, inter alia, resveratrol has been introduced (Drygalski et al., 2018).

According to previous research, the clinical use of resveratrol is considerably limited due to the low oral bioavailability caused by the short biological half-life, poor water solubility, and rapid metabolism (Palle and Neerati, 2017; Peng et al., 2018). Therefore, studies are conducted to achieve an effective serum concentration of this polyphenol to reach many of the proposed sites of action outside the gastrointestinal (GI) tract after an oral dose of 25 mg to even 5 g (Peng et al., 2018; Wang and Sang, 2018). Moreover, efforts have been made to modify resveratrol for improved bioavailability and reduced toxicity (Smoliga and Blanchard, 2014). The process of micronization was used to enhance resveratrol absorption across the gastrointestinal tract (Vesely et al., 2021). One of the most interesting aspects of its future development as a promising drug is that resveratrol does not appear to have side effects at short-term dosages, and no major side effects have been found in long-term clinical trials. At doses of 2.5 g or more per day, side effects such as nausea, vomiting, and diarrhea may occur (Salehi et al., 2018; Shaito et al., 2020). However, thus far little attention has been given to the relaxing effect of the human GI smooth muscle caused by resveratrol.

GI motility is a particularly important and complex physiological function of the digestive tract, regulated by many factors. The main factor responsible for GI symptoms is gastric mobility dysfunction. Sex hormones, estrogens, in particular, are known to cause GI motility disorder and contribute to irritable bowel syndrome (Zhang et al., 2014). Resveratrol is structurally and functionally similar to estrogens and belongs to the group of phytoestrogens. Recent data indicate that resveratrol may inhibit the contractility of the GI tract in rats and guinea pigs (Zhang et al., 2014). Despite a large number of studies on the beneficial effects of resveratrol on smooth muscles, the underlying mechanisms are not fully understood. The relaxation of GI smooth muscles is controlled by non-adrenergic non-cholinergic (NANC) signaling regulated by neurons of the myenteric plexus located between the circular and longitudinal muscle layers (Van Geldre and Lefebvre, 2004). One of the main NANC mediators is nitric oxide (NO), a gaseous neurotransmitter synthesized from L-arginine by nitric oxide synthase (NOS) in response to neuronal stimulation (Bult et al., 1990). NO increases the cellular cGMP level, which leads to the K^+^-channel activation: high conductance Ca^2+^-dependent (BK_Ca_) and small conductance apamin-sensitive (SK_Ca_) (Matsuda and Miller, 2009). Furthermore, direct activation of cGMP-independent K^+^-channels, as well as inhibition L-type Ca^2+^-channels, is mediated by the NO pathway (Matsuda and Miller, 2009). All these molecular mechanisms lead to hyperpolarization of the cell membrane resulting in smooth muscle relaxation.

Resveratrol can modify nitric oxide (NO) levels by its action on both endothelial NOS and cytokine-inducible NOS (Kline and Karpinski, 2015). Moreover, resveratrol exerts both indirect and direct vasodilator effects on blood vessels by NO-mediated and non-NO-mediated mechanisms (Choi et al., 2016). Furthermore, only a few studies of the effects of resveratrol in the gastrointestinal smooth muscle have been conducted and its molecular mechanism of action remains unclear.

Our study aimed to investigate the role of nitric oxide and potassium channels in resveratrol-induced relaxation of human gastric smooth muscle. Furthermore, the results of our preclinical research may become the basis for further extensive clinical studies with resveratrol in the treatment of gastrointestinal dyspepsia and other gastric hypermotility disorders.

## Materials and Methods

### Sample Processing for Isometric Contraction

**FIGURE 1:**
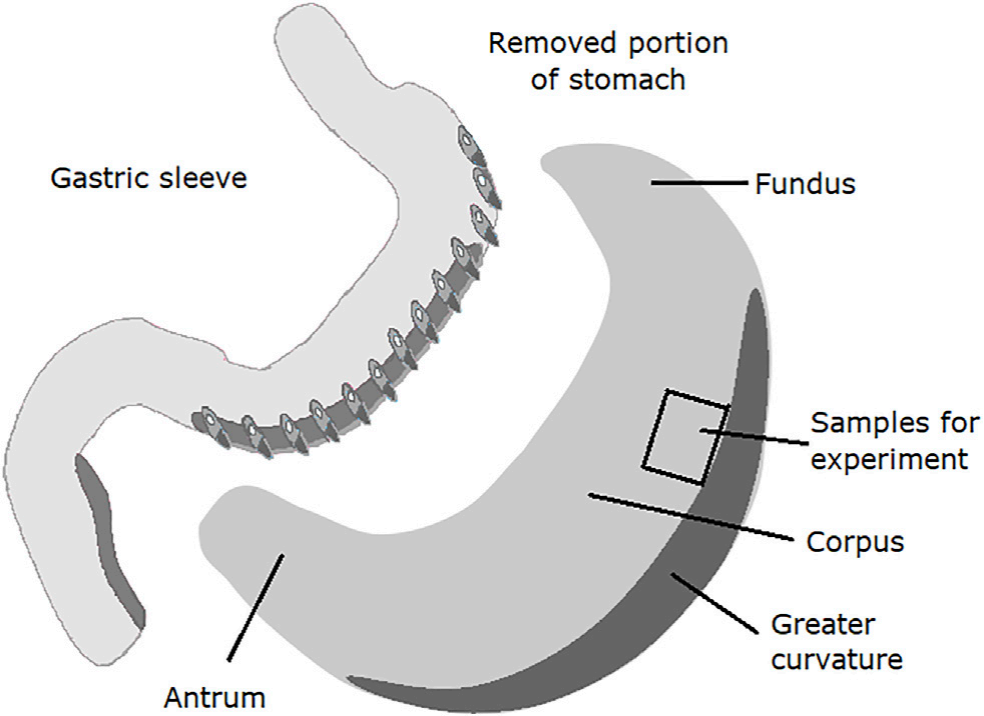
Diagram of anatomical areas of the stomach after gastrectomy using the vertical sleeve method. The place of sampling for the conducted experiments was marked.

The study was conducted under the Helsinki Declaration principles, the International Conference on Harmonization Guideline for Good Clinical Practice, the laws and regulations of Poland, and with the approval from the local Ethical Committee (No. R-I-002/304/2018). Human gastric tissues were obtained from patients who underwent sleeve gastrectomy for morbid obesity (*n* = 10 aged 21–48; BMI 48.21 ± 1.14). The collection of the tissues did not interfere with the surgical procedure. Samples were taken from the upper half of the stomach with larger curvature removed during the surgical procedure (Figure 1) (Hady et al., 2012). All patients were carefully informed about the aim and nature of the study before surgery and signed written consent.

All surgeries were performed under general anesthesia performed by the same team of anesthesiologists. Combined general anesthesia was induced by propofol (1.0–1.5 mg per kg body mass) and opioid analgesic fentanyl (1.0–1.5 μg per kg body mass). A non-depolarizing neuromuscular blocking agent cisatrocurium (0.1–0.2 mg per kg body mass) was also administered at that stage. Maintenance of general anesthesia was achieved with the volatile method with sevoflurane administered in repetitive doses. Additional doses of cisatrocurium and opioids were also administered depending on the patient’s needs and metabolism.

After removal, specimens were immediately cooled on ice-cold Tyrode’s buffer, bubbled with carbogen (95% O_2_ +5% CO_2_), and transported to the laboratory, and then treated as previously described (Pedzińska-Betiuk et al., 2011). Subsequently, the muscle layer was dissected from the gastric wall and cut in the direction of the longitudinal muscles into 10 mm × 3 mm × 1.5 mm strips. The tissues were attached to an isometric force transducer and placed in 20 ml tissue bath chambers. The bath temperature was kept constantly at 37°C and the carbogen was continuously bubbled. The preparations were allowed to equilibrate for 1 h. During this period the passive tension was adjusted to 2 mN and the organ bath solution was exchanged every 20 min. Before each experiment, strips were activated by 80 mmol/L K^+^. Only strips showing a stable response to potassium were used in the experiments (Modzelewska et al., 2019; Modzelewska et al., 2017).

### Experimental Protocol

After the equilibration period, contractile activity was stimulated using carbachol (10^–6^ mol/L) and taken as control after reaching its plateau. Then, resveratrol was added cumulatively to the organ chambers in the range from 10^−7^ to 10^–4^ mol/L every 10 min. To eliminate the effects of the resveratrol solvent, the influence of ethanol alone at the same concentration on gastric strips was investigated. The solvent did not influence the tone of the tissues, the responses to the relaxant agent were reproducible in the control strips unless otherwise stated. Furthermore, NOS synthase blockers (L-NNA, L-NAME—both 10^–5^ mol/L) and a soluble guanylate cyclase blocker (ODQ—10^–6^ mol/L) were used to establish the role of NOS in the relaxing effect of resveratrol. Additionally, concentration-response curves for resveratrol were constructed in the absence and presence of various potassium channels blockers to investigate their involvement. A non-selective K^+^ channel blocker—tetraethylammonium chloride (TEA—10^–3^ mol/L), a selective inhibitor of BK_Ca_—10^–7^ mol/L iberiotoxin (IbTX), an inhibitor of BK_Ca_, and intermediate (IK_Ca_) conductance calcium-activated potassium channels (K_Ca_), and slowly inactivating voltage-gated K_Ca_—10^–7^ mol/L charybdotoxin (ChTX), an SK_Ca_ blocker—10^–6^ mol/L apamin, a K_V_ channel blocker—10^–3^ mol/L 4-Aminopiridine (4AP) or a K_ATP_ channel blocker—10^–6^ mol/L glibenclamide, and a selective estrogen response modifier, and protein kinase C inhibitor—10^–6^ mol/L tamoxifen were added 20 min before the addition of resveratrol. Whenever possible, experiments were conducted with tissues obtained from the same patient and were tested in parallel. In a separate series of experiments, controls were performed under comparable conditions of the experiment and at the same time.

### Contraction Measurements

Data acquisition was performed by an isometric force transducer (BIO-SYS-TECH, Bialystok, Poland). The following parameters such as the area under the curve (AUC), average baseline muscle tone, and relative change in muscle contraction were evaluated with the DASYLab software (version 9.0; Laboratory Data Acquisition System, SuperLogics, Waltham, MA, United States). The results were presented as the stress–stretch ratio. All outcomes from two to four strips from each sample were averaged at the 10-min interval for each dose of the substance used. The AUC revealed the contractile activity of examined sample responses before and after the administration of the given drug. (Gagnon and Peterson, 1998; Modzelewska et al., 2003). The values of the AUC were assessed by calculating the integral of the suitable 10-min interval of the curve. Concentration-response curves were fitted to the logistic equation using nonlinear regression Y=Bottom+(Top-Bottom)/(1 + 10^((LogEC50-X)*HillSlope)) (PRISM 6.0, GraphPad Software Inc., San Diego, CA, United States). The maximum relaxing response (E_max_) was presented as a percentage of the values obtained just before the addition of the test substance. Subsequently, the concentrations of a compound where 50% of its maximal effect is observed were expressed as -log EC_50_. The values were demonstrated as the standard error of the mean (±SEM) of sample data performed on strips from various patients or as the percentage change [(effect size—baseline contractile response)/baseline contractile response × 100]. Results from pairs of responses were averaged.

### Chemicals

Resveratrol (5-[(1*E*)-2-(4-Hydroxyphenyl)ethenyl]-1,3,benzenediol) was purchased from abcr GmbH (Karlsruhe, Germany). Carbamylcholine chloride ((2-Hydroxyethyl)trimethylammonium chloride carbamate; carbachol), N^G^-Methyl-L-Arg (Nω-Nitro-L-arginine methyl ester hydrochloride, L-NAME), N5-(Nitroamidino)-L-2,5-diaminopentanoic acid, N^G^-NO_2_-L-Arg (N_ω_-Nitro-L-arginine, L-NNA), 1H-[1,2,4]Oxadiazolo[4,3-a]quinoxalin-1-one (ODQ), iberiotoxin (IbTX), charybdotoxin (ChTX), apamin, glibenclamide, N,N,N,N-Tetraethylammonium chloride (TEA), 4-Aminopiridine (4AP) and tamoxifen were purchased from the Sigma Chemical Company, were purchased from Sigma (St. Louis, MO).

Resveratrol was dissolved in 70% ethanol so that the final concentration of ethanol was never >0.1%, which did not affect basal contraction. The working solutions were prepared fresh on the day of the experiment by diluting the stock solution.

Stock solutions of carbachol, L-NNA, L-NAME, apamin, IbTX, ChTX, TEA, 4AP, and tamoxifen were prepared with bidistilled water, and glibenclamide and ODQ were dissolved in dimethyl sulphoxide (DMSO). The given concentrations were the calculated final concentrations in the organ bath solution. All reagents were added directly to the bath fluid containing a Tyrode’s solution composed of (mmol/L): NaCl 139.6; KCl 2.68; MgCl_2_ 1.05; NaH_2_PO_4_ 1.33; CaCl_2_ 1.80; NaHCO_3_ 25.0; and glucose 5.55.

### Statistical Analysis

Statistical analysis was performed using GraphPad Prism 6.0 (GraphPad Software Inc., San Diego, United States). The DʼAgostino-Pearson test was used to determine the normal distribution of the investigated variables, and then the compliance with the Gaussian distribution was checked. For comparing values of the two following measurements, the one-way ANOVA or the Kruskal-Wallis test was used, where appropriate. Statistically significant differences between means were determined by Tukey’s *post-hoc* or a nonparametric Mann-Whitney *U* test, where appropriate. A probability value of less than 0.05 was regarded as significant.

## Results

**FIGURE 2:**
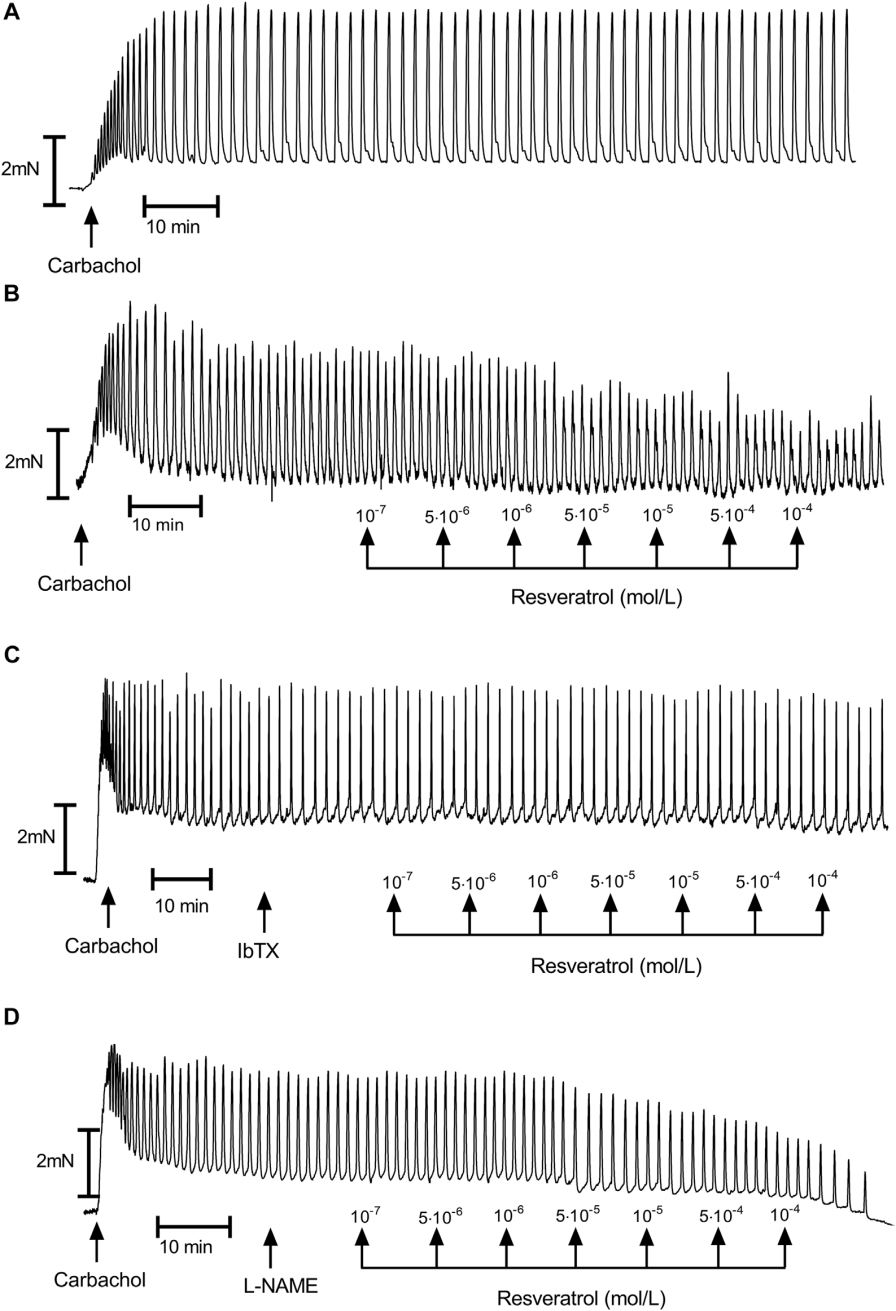
A representative tracings of carbachol-induced gastric muscle contractions. **(A)**—control; **(B)**—resveratrol (range 10^–7^–10^–4^ mol/L); **(C)**—resveratrol (range 10^–7^–10^–4^ mol/L) after preincubation with iberiotoxin (10^–7^ mol/L); **(D)**—resveratrol (range 10^–7^–10^–4^ mol/L) after preincubation with L-NAME (10^–6^ mol/L).

Carbachol (10^–6^ mol/L) promoted noticeable and long-duration contractions in muscle strips isolated from the upper half of the human stomach (Figures 1, 2). Typical tracings show the response of gastric smooth muscle strips to cumulatively applied resveratrol (Figure 2).

**FIGURE 3:**
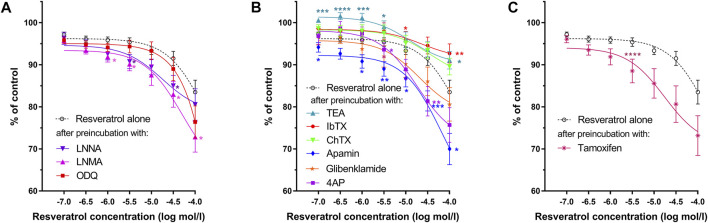
Effects of resveratrol on the gastric muscle samples as measured by AUC after preincubation with **(A)**—L-NNA, L-NAME, ODQ; **(B)**—TEA, IbTX, ChTX, apamin glibenclamide or 4AP; **(C)**—tamoxifen. Each point represents the mean ± SEM of AUC values obtained from individual strips (*n* = 10) from different patients. AUC of carbachol-induced contractions of each gastric strip over a 10-min interval before the addition of the antagonist was treated as control. Significance at *p < 0.05; **p < 0.01; ***p < 0.001; ****p < 0.0001 as compared to the AUC of resveratrol alone.

**FIGURE 4:**
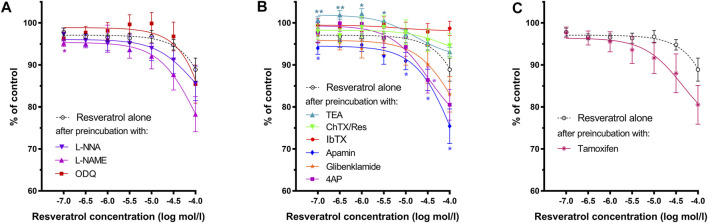
Effects of resveratrol on the basal tension of gastric muscle samples after preincubation with **(A)**—L-NNA, L-NAME, ODQ; **(B)**—TEA, IbTX, ChTX, apamin glibenclamide or 4AP; **(C)**—tamoxifen. Each point represents the mean ± SEM of basal tension values obtained from individual strips (*n* = 10) from different patients. Basal tension of carbachol-induced contractions of each gastric strip over a 10-min interval before the addition of the antagonist was treated as control. Significance at *p < 0.05; **p < 0.01 as compared to the basal tension of resveratrol alone.

Resveratrol induced a definite, dose-dependent relaxation of the gastric strips. (Figures 2B, 3, 4), substantial—at 10^–7^ mol/L and maximal—at 10^–4^ mol/L. The maximal response caused by 10^–4^ mol/L resveratrol was extremely significant—83.49 ± 2.85% (*n* = 10; *p* = 0.0003) of the contractions of the strips before resveratrol administration (Figure 3 and Table 1 and Supplementary Table S1). Resveratrol dose-dependently reduced baseline tension at all concentrations. The reduction was considerably lowered the resting tension only in 5 × 10^–6^ and 10^–4^ mol/L (Figure 4 and Table 1 and Supplementary Table S2).

**TABLE 1:** Log EC_50_ and E_max_ for resveratrol on carbachol-induced contractility of the human gastric muscles. The values are mean ± SD of *n* = 10 individual gastric strips from different patients. *p < 0.05, **p < 0.01, ***p < 0.001, ****p < 0.0001 versus resveratrol alone; ^¥^ *p* < 0.05 versus preincubation with IbTX.

	logEC_50_	*p*	E_max_	*p*
Resveratrol alone	−3.75 ± 0.19		83.49 ± 2.85	
Resveratrol after preincubation with				
L-NNA	−4.75 ± 0.11***	*p* < 0.001	80.55 ± 3.75	0.610
L-NAME	−4.29 ± 0.09*	0.020	72.84 ± 3.64	0.040
ODQ	−2.46 ± 2.75	0.640	76.40 ± 3.82	0.070
TEA	−4.90 ± 0.29****	*p* < 0.0001	91.08 ± 2.07*	*p* < 0.05
IbTX	−3.72 ± 2.97	0.980	95.60 ± 1.52**	*p* < 0.010
ChTX	−4.44 ± 0.48*	0.011	89.58 ± 1.98^¥^	0.040
Apamin	−4.18 ± 0.09	0.056	69.96 ± 3.72*	0.010
Glibenclamide	−4.69 ± 0.13***	*p* < 0.001	80.59 ± 3.97	0.490
4AP	−4,74 ± 0.25***	*p* < 0.001	75.68 ± 4.13	*p* = 0.09
Tamoxifen	−4.76 ± 0.10***	*p* < 0.001	73.17 ± 4.74	0.138

As shown in Figure 3A and Table 1, both NOS (L-NNA, L-NAME) blockers and a guanylate cyclase blocker (ODQ) did not affect the course of the relaxing response of the samples induced by resveratrol (*n* = 10). Also, when the basal tension changes were observed, no clear effect of preincubation with the above blockers was observed. (Figures 2D, 4A). Yet, we observed a statistically significant shift to the left of the concentration-response curve for resveratrol after preincubation with L-NNA (*p* < 0.001) or L-NAME (*p* < 0.05) compared with the experiments without NOS blockers (Table 1). These results indicate that resveratrol-induced relaxation does not involve the activation of the nitric oxide pathway.

Conversely, ChTX (*n* = 10) tended to inhibit resveratrol-induced relaxation. Preincubation with IbTX inhibited the relaxing effect of resveratrol, which was statistically significant at concentrations higher than 5 × 10^–5^ mol/L (Figures 2C, 3, 4). A non-selective K^+^ channel blocker—TEA (*n* = 10), as well as selective BK_Ca_ channel blockers (IbTX and ChTX), reduced the relaxant effects of resveratrol, which was extremely significant in low resveratrol concentrations and statistically significant in the highest concentration of the AUC values, and between basal tension values below 10^–5^ mol/L (Figure 3B; Table 1 and Supplementary Table S1).

Apamin (*n* = 10) enhanced the relaxing effect of the concentration-response curve of resveratrol. The difference between AUC values was substantial at resveratrol concentrations above 10^–6^ mol/L (extremely at 5 × 10^–4^ mol/L) and between basal tension values above 5 × 10^–5^ mol/L (Figures 3B, 4B; Table 1, Supplementary Table S1, S2). Preincubation with glibenclamide (n = 10) did not significantly alter the relaxing effect of resveratrol on AUC and basal tension. However, there was an extremely significant left shift from the concentration-response curve for resveratrol (*p* < 0.001) (Figures 3B, 4B and Table 1, Supplementary Table S1, S2).

Blocking K_v_ -dependent K^+^ channels by 4AP (*n* = 10) enhanced the relaxant effect of resveratrol, statistically significant in almost all resveratrol concentrations (Figure 3B and Table 1, and Supplementary Table S1). Preincubation with 4AP lowered the basal tension compared with the experiments without 4AP (Figure 4B and Table 1, and Supplementary Table S2) The effect was statistically substantial in the lowest and higher than 5 × 10^–5^ mol/L concentrations.

Incubation of gastric smooth muscle strips with tamoxifen enhanced resveratrol-induced relaxation, extremely substantially at 5 × 10^–5^ mol/L (AUC) without significant influence on their basal tension (Figures 3C, 4C, Supplementary Table S1). For tamoxifen, also a statistically substantial shift to the left of the concentration-response curve for resveratrol was observed (*p* < 0.001) (Table 1).

## Discussion

Resveratrol has been shown to relax various types of smooth muscle, including arteries (Naderali et al., 2001; Shen et al., 2013; Choi et al., 2016; Breuss et al., 2019), umbilical vein (Hassanpour et al., 2021), uterus (Wu et al., 2015), gallbladder (Kline and Karpinski, 2015), corpus cavernosum (Soner et al., 2010) and GI tract (Zhang et al., 2014; Parlar and Arslan, 2019; Modzelewska et al., 2021). In previous studies, doses were used in a fairly wide range, and due to the low oral bioavailability of resveratrol, an orally administered nutraceutical operated almost exclusively in the gastrointestinal tract (Palle and Neerati, 2017; Peng et al., 2018). Considering the above, we decided to examine the influence of this polyphenol on the human gastric muscles contractions *in vitro*.

We found resveratrol concentration-dependently decreased human gastric muscle contractions in all concentrations used. Our finding is consistent with data reported for the influence of flavonoids on GI muscles (Zhang et al., 2014; Modzelewska et al., 2021).

Due to the structural similarity between resveratrol and synthetic estrogen diethylstilbestrol (Zhang et al., 2014), we investigated whether resveratrol might exhibit an estrogenic effect on GI motility. However, the results of the present study show that this is unlikely, as the relaxing effect of resveratrol was not inhibited by tamoxifen. On the contrary, tamoxifen tended to enhance resveratrol-induced relaxation. A statistically significant left shift in the concentration-response curve can be attributed to the fact that resveratrol acts as an agonist at the estrogen GPER-1 receptor (Levenson et al., 2003). Hence, in the control group, some resveratrol molecules are bound to these receptors. When incubated with tamoxifen, the resveratrol binding may have become blocked. Therefore, compared to control, a greater amount of resveratrol particles was available to interact with BK_Ca_ channels, thus enhancing relaxation.

The ability of NO to relax smooth muscles is well established (Modzelewska et al., 2019, Modzelewska et al., 2021; Idrizaj et al., 2021). One of the mechanisms of NO smooth muscle relaxation is the activation of soluble guanylyl cyclase (sGC) (Friebe et al., 2018), which is expressed in a variety of cell types and signals through cGMP on to cGMP-dependent protein kinase (PKG), phosphodiesterases or, possibly, on to cGMP-regulated channels (Friebe and Koesling, 2003). Previous research has demonstrated that resveratrol-induced smooth muscle relaxation was primarily related to NO and potassium channels. (Choi et al., 2016). The NO pathway *via* cGMP-dependent or independent mechanisms has been reported to be involved in the regulation of the muscle tone of the GI relaxation response. (Rocha et al., 2014). Therefore, the influence of the NO pathway on the mechanism of action of polyphenols in the GI is not clearly defined. Tsai et al. demonstrated that the resveratrol-induced relaxation of the guinea pig fundus was inhibited by L-NNA, indicating that this process is mediated by both the NOS of the neuron or the smooth muscle of the fundus (Tsai et al., 2018). However, Zhang et al. presented that resveratrol relaxes GI smooth muscle partially by nitrergic pathway (Zhang et al., 2014). In turn, Amira et al. showed the inhibitory effect of flavonoids on the gastric tone in mouse isolated stomach strips was not significantly reduced in the presence of L-NAME, suggesting that it is not related to NO production (Amira et al., 2008).

In the present study, we demonstrated that resveratrol-induced relaxation was not inhibited by the NOS inhibitor L-NNA or L-NAME, and the sGC inhibitor ODQ, as well. The -log EC_50_ for the effect of resveratrol after preincubation with L-NNA (*p* < 0.001) or L-NAME (*p* < 0.05) showed a statistically significant shift to the left versus a curve without NOS blockers. In our experiment, blocking soluble guanylyl cyclase (sGC) by ODQ virtually did not change the relaxing effect of resveratrol. Therefore, it is possible that blocking the activation of the NO pathway can enhance the relaxant effects of resveratrol of the human gastric smooth muscle and may indicate the complexity of the process of calcium release from the sarcoplasmic reticulum. It can be hypothesized that this process, which may be mediated by resveratrol or NO, is not synergistic but competitive. This could further support the leading conclusion of our research that the main mechanism of resveratrol triggering the relaxation of gastric smooth muscle is the activation of Ca^2+^-dependent K^+^ channels. This preliminary hypothesis requires more in-depth analysis and experimental confirmation. Presented data is in line with our previous results for quercetin. There, too, no effect of blocking the NO pathway on gastric muscle relaxation was observed. (Modzelewska et al., 2021). Thus, our findings indicate that resveratrol can cause the human gastric muscles to relax independently of the NO pathway. On the other hand, different contractile stimulants and species may influence the difference in resveratrol-induced relaxation mechanisms between rats, guinea pigs, and human GI smooth muscle. Thus, more research is needed to elucidate the difference between the different species.

Since neither L-NNA, L-NAME nor ODQ inhibited resveratrol-induced relaxation of human gastric strips, we investigated the effect of blocking various types of K^+^ on this effect in subsequent experiments. Activation of K^+^ channels of the cell membrane causes its hyperpolarization and additionally suppresses the influx of Ca^2+^ into the cell, causing relaxation of smooth muscles. Apamin, SK_Ca_ channels blocker as well as glibenclamide (K_ATP_ channels blocker) did not inhibit the relaxing effect of resveratrol. However, there was a statistically significant left shift from the -logEC_50_ curve for resveratrol (*p* < 0.001) after preincubation with glibenclamide. This raises questions as to the mechanism of such enhancement of the relaxing effect of resveratrol. When we block K_ATP_ channels, in addition to inhibiting the outflow of K^+^ ions from the cell, the membrane potential also changes (Brayden, 2002). Under their regulation, activation of voltage-gated calcium channels is possible, calcium influx, and possibly additionally activation of Ca^2+^-dependent K^+^ channels. The verification of this hypothesis requires further research. Instead, blocking BK_Ca_ by a specific blocker of these channels IbTX resulted in complete inhibition of resveratrol-induced relaxation. ChTX also inhibited the effect of resveratrol, but this effect was weaker than that of IbTX. This is probably because ChTX is a non-specific BK_Ca_ channel blocker acting simultaneously on the IK_Ca_ channels and slowly inactivating voltage-gated K^+^ channels (Kv1.3) (Giangiacomo et al., 1993). Tan et al., 2020 demonstrated that resveratrol targets multiple signaling pathways to exert a vasorelaxant effect in a rat aortic ring model specifically can suppress not only extracellular calcium influx but also an intracellular release of calcium from the sarcoplasmic reticulum within vascular smooth muscle cells (Tan et al., 2020). Then it should be considered, what if resveratrol works by inhibiting Ca^2+^ channels? The consequence of Ca^2+^ channel inhibition is a subsequent decrease in Ca^2+^ concentration inside the cell, followed by muscle relaxation. However, at the same time, Ca^2+^-dependent K^+^ channels remain closed, because the factor that causes them to open is an increase in the Ca^2+^ concentration inside the cell (Borowiec et al., 2014). Therefore, blocking them by iberiotoxin, charybdotoxin or TEA should not affect their activity, and thus the strength of muscle contraction. In our experiment, we demonstrated that blocking these Ca^2+^-dependent K^+^ channels abolished the relaxing effects of resveratrol. Hence, we can conclude that resveratrol acts either directly on BK_Ca_ channels to open them or acts locally to release small amounts of Ca^2+^ from the sarcoplasmic reticulum which is in contrast to the Tan et al., 2020 findings. BK_Ca_ channels are considered channel complexes formed by an ion-conducting α-subunit and regulatory β (β_1-4_)- or γ (γ_1-4_)-subunits (Sancho and Kyle, 2021). They have been reported to be modulated by depolarization, calcium, stretch-triggered, independently and even if the Ca^2+^ channel is inhibited, BK_Ca_ may still be activated (Qi et al., 2005; Wang et al., 2010; Xin et al., 2018). However, little is known about the mechanisms underlying the activation and termination of Ca^2+^ sparks in muscle (Fill and Copello, 2002; Vesely et al., 2021). Moreover, the detailed molecular mechanism that activates BK_Ca_ channels by membrane stretch remains unclear, nevertheless, the modulating role is attributed to the β_1_-subunit of the BK_Ca_ channel (Xin et al., 2018). Therefore, in our research, we used ChTX and the highly selective IbTX, which act as pore blockers, occluding conduction pathways of the α-subunit, and are useful experimental tools, mainly due to their poor reversibility and exclusion of the influence of other units. Additionally, a quaternary amine, such as TEA blocks BK_Ca_ channels through either the internal or external side of the membrane, implying a complex mechanism of action in a voltage-dependent manner (Contreras et al., 2013; Sancho and Kyle, 2021). Hence, our results support the idea that resveratrol induces the relaxation of the smooth muscles of the human stomach through the activation of BK_Ca_ channels.

Few studies have been published explaining resveratrol-induced smooth muscle relaxation, and those that exist propose different pathways of action of this polyphenol. Moreover, they are mainly animal studies. Tsai et al. have shown that resveratrol-induced relaxation of the guinea pig fundus occurs through the NO pathway and K_ATP_ channels (Tsai et al., 2018). In turn, Zhang et al. reached a different conclusion by studying rat gastrointestinal smooth muscle. Their results indicate that resveratrol relaxes rat’s gastrointestinal smooth muscle *via* α-adrenergic receptors, NO and cyclic adenosine monophosphate pathways, K_ATP_ channels, and inhibition of L-type Ca^2+^channels (Zhang et al., 2012). Several papers also describe the relaxant effects of this polyphenol on rat arteries. Gojkovic-Bukarica et al. showed that this process involves activation voltage-gated K^+^ channels (Novakovic et al., 2006; Gojkovic-Bukarica et al., 2008). The same mechanism is also indicated by the same authors for the relaxation of the rat renal artery (Gojkovic-Bukarica et al., 2019). In turn, Dalaklioglu and Ozbey have shown that 4AP did not significantly alter relaxant responses of rat corpus cavernosum strips to resveratrol (Dalaklioglu and Ozbey, 2014). In our experiments, concentration-response relationships of resveratrol and 4AP, a non-specific blocker of K_V_ channels, have shown that K_V_ channels do not participate in the relaxing effect of resveratrol on human gastric smooth muscle. Membrane depolarization activates K_V_ channels, and, in general, they participate in the negative feedback regulation of muscle contraction along with BK_Ca_ channels. Consistent with this negative-feedback role, block of K_V_ channels potentiates contraction induced by vasoconstrictors (Jackson, 2017). And since, rather than contraction, an enhancement of resveratrol-induced relaxation is observed, it could be suggested that the process is competitive rather than synergistic. On the other hand, Chen et al., also examining the rat aorta, indicated a different, by NO-mediated mechanism of relaxation (Chen and Pace-Asciak, 1996). Regarding the *in vitro* studies of human smooth muscles, these concerned the evaluation of the relaxing effect of resveratrol on the muscles of the gallbladder. The results of the above research show that inhibition of contractions is associated with NO, K_ATP_ channels, and BK_Ca_ channel pathways (Tsai et al., 2017). While all authors agree that resveratrol has a relaxant effect on smooth muscles, it can be noted that the proposed mechanisms describing this process differ considerably. Importantly, the muscle relaxant effect of resveratrol, despite its low bioavailability, may partly explain its influence on the oral glucose tolerance test in diabetic studies (Jakubczyk et al., 2020). The reduction of GI tract motility lowers the stomach emptying rate what affects the pharmacokinetics of glucose or digested meals preventing postprandial hyperglycemia. This opens the way for further research of this compound both at the level of pharmacokinetics and its molecular effects in tissues.

Dietary resveratrol and/or its metabolites, as they pass along the GI tract, possess a wide spectrum of valuable local physiological and pharmacological effects. On a systemic level, its absorption may exhibit a wide range of beneficial properties. The impact of regional differences in anatomy and physiology across the GI tract on drug absorption is significant and for some classes of active pharmaceutical ingredients, such as orally administered polyphenols, it is a challenge to understand the nature of these barriers. Accordingly, the beneficial effects of these compounds, including resveratrol, may occur *in situ*. Moreover, in the results of clinical trials to date, there have been no reports of the relaxing properties of resveratrol in the gastrointestinal tract. (Salehi et al., 2018; Singh et al., 2019; Shaito et al., 2020). This indicates that it may be beneficial to conduct a study focusing on the relaxant properties of resveratrol in gastrointestinal disorders. Therefore, it is worth researching resveratrol as well as other nutraceuticals that increase gastric muscle relaxation as a potential drug to functional dyspepsia.

## Conclusion

In conclusion, this study provides the first observation that the relaxant effects of resveratrol in human gastric muscle strips occur directly through BK_Ca_ channels and independently of NO pathways. Furthermore, there is considerable potential for further extensive clinical studies with resveratrol as an effective new drug or health supplement to treat gastrointestinal dyspepsia and other gastric hypermotility disorders.

## Data Availability

The raw data supporting the conclusion of this article will be made available by the authors, without undue reservation.

## References

[B1] Amira S. Rotondo A. Mulè F. (2008). Relaxant Effects of Flavonoids on the Mouse Isolated Stomach: Structure-Activity Relationships. Eur. J. Pharmacol. 599, 126–130. 10.1016/j.ejphar.2008.09.021 18840426

[B2] Berk K. Drygalski K. Harasim-Symbor E. Charytoniuk T. Iłowska N. Łukaszuk B. (2019). The Effect of Enterolactone on Liver Lipid Precursors of Inflammation. Life Sci. 221, 341–347. 10.1016/j.lfs.2019.02.044 30802511

[B3] Borowiec A. S. Bidaux G. Pigat N. Goffin V. Bernichtein S. Capiod T. (2014). Calcium Channels, External Calcium Concentration and Cell Proliferation. Eur. J. Pharmacol. 739, 19–25. 10.1016/j.ejphar.2013.10.072 24291106

[B4] Brayden J. E. (2002). Functional Roles of KATP Channels in Vascular Smooth Muscle. Clin. Exp. Pharmacol. Physiol. 29, 312–316. 10.1046/j.1440-1681.2002.03650.x 11985542

[B5] Breuss J. M. Atanasov A. G. Uhrin P. (2019). Resveratrol and its Effects on the Vascular System. Int. J. Mol. Sci. 20, 1523. 10.3390/ijms20071523 PMC6479680 30934670

[B6] Bult H. Boeckxstaens G. E. Pelckmans P. A. Jordaens F. H. Van Maercke Y. M. Herman A. G. (1990). Nitric Oxide as an Inhibitory Non-adrenergic Non-cholinergic Neurotransmitter. Nature 345, 346–347. 10.1038/345346a0 1971425

[B7] Charytoniuk T. Harasim-Symbor E. Polak A. Drygalski K. Berk K. Chabowski A. (2018). Influence of Resveratrol on Sphingolipid Metabolism in Hepatocellular Carcinoma Cells in Lipid Overload State. Anticancer. Agents Med. Chem. 19, 121–129. 10.2174/1871520619666181224161255 30585550

[B8] Chen C. K. Pace-Asciak C. R. (1996). Vasorelaxing Activity of Resveratrol and Quercetin in Isolated Rat Aorta. Gen. Pharmacol. 27, 363–366. 10.1016/0306-3623(95)02001-2 8919657

[B9] Choi S. Ryu K. H. Park S. H. Jun J. Y. Shin B. C. Chung J. H. (2016). Direct Vascular Actions of Quercetin in Aorta from Renal Hypertensive Rats. Kidney Res. Clin. Pract. 35, 15–21. 10.1016/j.krcp.2015.12.003 27069853 PMC4811985

[B54] Contreras G. F. Castillo K. Enrique N. Carrasquel-Ursulaez W. Castillo J. P. Milesi V. (2013). A BK (Slo1) Channel Journey From Molecule to Physiology. Channels 7, 442. 10.4161/CHAN.26242 24025517 PMC4042479

[B10] Dalaklioglu S. Ozbey G. (2014). Role of Different Types of Potassium Channels in the Relaxation of Corpus Cavernosum Induced by Resveratrol. Pharmacogn. Mag. 10, 47–52. 10.4103/0973-1296.126658 24696545 PMC3969658

[B11] Drygalski K. Fereniec E. Koryciński K. Chomentowski A. Kiełczewska A. Odrzygóźdź C. (2018). Resveratrol and Alzheimer's Disease. From Molecular Pathophysiology to Clinical Trials. Exp. Gerontol. 113, 36–47. 10.1016/J.EXGER.2018.09.019 30266470

[B12] Fill M. Copello J. A. (2002). Ryanodine Receptor Calcium Release Channels. Physiol. Rev. 82, 893–922. 10.1152/physrev.00013.2002 12270947

[B13] Friebe A. Koesling D. (2003). Regulation of Nitric Oxide-Sensitive Guanylyl Cyclase. Circ. Res. 93, 96–105. 10.1161/01.RES.0000082524.34487.31 12881475

[B14] Friebe A. Voußen B. Groneberg D. (2018). NO-GC in Cells 'off the Beaten Track'. Nitric Oxide 77, 12–18. 10.1016/j.niox.2018.03.020 29626542

[B15] Gagnon R. C. Peterson J. J. (1998). Estimation of Confidence Intervals for Area under the Curve from Destructively Obtained Pharmacokinetic Data. J. Pharmacokinet. Biopharm. 26, 87–102. 10.1023/A:1023228925137 9773394

[B16] Giangiacomo K. M. Sugg E. E. Garcia-Calvo M. Leonard R. J. McManus O. B. Kaczorowski G. J. (1993). Synthetic Charybdotoxin-Iberiotoxin Chimeric Peptides Define Toxin Binding Sites on Calcium-Activated and Voltage-dependent Potassium Channels. Biochemistry 32, 2363–2370. 10.1021/bi00060a030 7680230

[B17] Gojkovic-Bukarica L. Novakovic A. Kanjuh V. Bumbasirevic M. Lesic A. Heinle H. (2008). A Role of Ion Channels in the Endothelium-independent Relaxation of Rat Mesenteric Artery Induced by Resveratrol. J. Pharmacol. Sci. 108, 124–130. 10.1254/jphs.08128FP 18818483

[B18] Gojkovic-Bukarica L. Markovic-Lipkovski J. Heinle H. Cirovic S. Rajkovic J. Djokic V. (2019). The Red Wine Polyphenol Resveratrol Induced Relaxation of the Isolated Renal Artery of Diabetic Rats: The Role of Potassium Channels. J. Funct. Foods 52, 266–275. 10.1016/j.jff.2018.11.009

[B19] Hady H. R. Dadan J. Gołaszewski P. (2012). 100 Obese Patients after Laparoscopic Adjustable Gastric Banding - the Influence on BMI, Gherlin and Insulin Concentration, Parameters of Lipid Balance and Co-morbidities. Adv. Med. Sci. 57, 58–64. 10.2478/v10039-012-0008-8 22440938

[B20] Hassanpour M. Biray Avci Ç. Rahbarghazi R. Rezabakhsh A. Nourazarian A. Nabat E. (2021). Resveratrol Reduced the Detrimental Effects of Malondialdehyde on Human Endothelial Cells. J. Cardiovasc. Thorac. Res. 13, 131–140. 10.34172/jcvtr.2021.27 34326967 PMC8302894

[B21] Idrizaj E. Traini C. Vannucchi M. G. Baccari M. C. (2021). Nitric Oxide: From Gastric Motility to Gastric Dysmotility. Ijms 22, 9990. 10.3390/ijms22189990 34576155 PMC8470306

[B22] Jackson W. F. (2017). Potassium Channels in Regulation of Vascular Smooth Muscle Contraction and Growth. Adv. Pharmacol. 78, 89–144. 10.1016/bs.apha.2016.07.001 28212804 PMC5518321

[B23] Jakubczyk K. Skonieczna-Żydecka K. Kałduńska J. Stachowska E. Gutowska I. Janda K. (2020). Effects of Resveratrol Supplementation in Patients with Non-alcoholic Fatty Liver Disease-A Meta-Analysis. Nutrients 12, 1–15. 10.3390/nu12082435 PMC7469003 32823621

[B24] Kline L. W. Karpinski E. (2015). The Resveratrol-Induced Relaxation of Cholecystokinin Octapeptide- or KCl-Induced Tension in Male Guinea Pig Gallbladder Strips Is Mediated through L-type Ca2+Channels. J. Neurogastroenterol. Motil. 21, 62–68. 10.5056/jnm14093 25537678 PMC4288087

[B25] Levenson A. S. Gehm B. D. Pearce S. T. Horiguchi J. Simons L. A. Ward J. E. (2003). Resveratrol Acts as an Estrogen Receptor (ER) Agonist in Breast Cancer Cells Stably Transfected with ER Alpha. Int. J. Cancer 104, 587–596. 10.1002/ijc.10992 12594813

[B26] Matsuda N. M. Miller S. M. (2009). Non-adrenergic Non-cholinergic Inhibition of Gastrointestinal Smooth Muscle and its Intracellular Mechanism(s). Fundam. Clin. Pharmacol. 24, 261–268. 10.1111/j.1472-8206.2009.00761.x 19674117

[B27] Modzelewska B. Jóźwik M. Jóźwik M. Sulkowski S. Pędzińska-Betiuk A. Kleszczewski T. (2017). Altered Uterine Contractility in Response to β-adrenoceptor Agonists in Ovarian Cancer. J. Physiol. Sci. 67, 711–722. 10.1007/s12576-016-0500-1 27838886 PMC5639028

[B28] Modzelewska B. Jóźwik M. Jóźwik M. Tylicka M. Kleszczewski T. (2019). The Effects of Extended Nitric Oxide Release on Responses of the Human Non-pregnant Myometrium to Endothelin-1 or Vasopressin. Pharmacol. Rep. 71, 892–898. 10.1016/j.pharep.2019.05.003 31419630

[B29] Modzelewska B. Kleszczewski T. Kostrzewska A. (2003). The Effect of a Selective Inhibition of Potassium Channels on the Relaxation Induced by Nitric Oxide in the Human Pregnant Myometrium. Cell. Mol. Biol. Lett. 8, 69–75. Available at: http://www.ncbi.nlm.nih.gov/pubmed/12655359 (Accessed May 18, 2016). 12655359

[B30] Modzelewska B. Drygalski K. Kleszczewski T. Chomentowski A. Koryciński K. Kiełczewska A. (2021). Quercetin Relaxes Human Gastric Smooth Muscles Directly through ATP‐sensitive Potassium Channels and Not Depending on the Nitric Oxide Pathway. Neurogastroenterology Motil. 33, e14093. 10.1111/nmo.14093 PMC8365708 33528064

[B31] Naderali E. K. Smith S. L. Doyle P. J. Williams G. (2001). The Mechanism of Resveratrol-Induced Vasorelaxation Differs in the Mesenteric Resistance Arteries of Lean and Obese Rats. Clin. Sci. (Lond) 100, 55–60. 10.1042/cs1000055 11115418

[B32] Novakovic A. Bukarica L. G. Kanjuh V. Heinle H. (2006). Potassium Channels-Mediated Vasorelaxation of Rat Aorta Induced by Resveratrol. Basic Clin. Pharmacol. Toxicol. 99, 360–364. 10.1111/j.1742-7843.2006.pto_531.x 17076688

[B33] Palle S. Neerati P. (2017). Enhancement of Oral Bioavailability of Rivastigmine with Quercetin Nanoparticles by Inhibiting CYP3A4 and Esterases. Pharmacol. Rep. 69, 365–370. 10.1016/j.pharep.2016.12.002 28189992

[B34] Parlar A. Arslan S. O. (2019). Resveratrol Normalizes the Deterioration of Smooth Muscle Contractility after Intestinal Ischemia and Reperfusion in Rats Associated with an Antioxidative Effect and Modulating Tumor Necrosis Factor Alpha Activity. Ann. Vasc. Surg. 61, 416–426. 10.1016/J.AVSG.2019.06.027 31449943

[B35] Pedzińska-Betiuk A. Modzelewska B. Jóźwik M. Kostrzewska A. (2011). Differences in the Effects of Beta2- and Beta3-Adrenoceptor Agonists on Spontaneous Contractions of Human Nonpregnant Myometrium. Ginekol. Pol. 82, 918–924. Available at: http://www.ncbi.nlm.nih.gov/pubmed/22384628 . 22384628

[B36] Peng R. M. Lin G. R. Ting Y. Hu J. Y. (2018). Oral Delivery System Enhanced the Bioavailability of Stilbenes: Resveratrol and Pterostilbene. BioFactors 44, 5–15. 10.1002/biof.1405 29322567

[B55] Qi Z. Chi S. Su X. Naruse K. Sokabe M. (2005). Activation of a Mechanosensitive BK Channel by Membrane Stress Created With Amphipaths. Mol. Membr. Biol. 22, 519–527. 10.1080/09687860500370703 16373323

[B37] Repossi G. Das U. N. Eynard A. R. (2020). Molecular Basis of the Beneficial Actions of Resveratrol. Arch. Med. Res. 51, 105–114. 10.1016/j.arcmed.2020.01.010 32111491

[B38] Rocha B. S. Nunes C. Pereira C. Barbosa R. M. Laranjinha J. (2014). A Shortcut to Wide-Ranging Biological Actions of Dietary Polyphenols: Modulation of the Nitrate-Nitrite-Nitric Oxide Pathway in the Gut. Food Funct. 5, 1646–1652. 10.1039/c4fo00124a 24912104

[B39] Salehi B. Mishra A. P. Nigam M. Sener B. Kilic M. Sharifi-Rad M. (2018). Resveratrol: A Double-Edged Sword in Health Benefits. Biomedicines 6. 10.3390/biomedicines6030091 PMC6164842 30205595

[B56] Sancho M. Kyle B. D. (2021). The Large-Conductance, Calcium-Activated Potassium Channel: A Big Key Regulator of Cell Physiology. Front. Physiol. 12, 1856. 10.3389/fphys.2021.750615 PMC8567177 34744788

[B40] Shaito A. Posadino A. M. Younes N. Hasan H. Halabi S. Alhababi D. (2020). Potential Adverse Effects of Resveratrol: A Literature Review. Int. J. Mol. Sci. 21, 2084. 10.3390/ijms21062084 PMC7139620 32197410

[B41] Shen M. Zhao L. Wu R. X. Yue S. Q. Pei J. M. (2013). The Vasorelaxing Effect of Resveratrol on Abdominal Aorta from Rats and its Underlying Mechanisms. Vascul. Pharmacol. 58, 64–70. 10.1016/j.vph.2012.07.005 22820258

[B42] Singh A. P. Singh R. Verma S. S. Rai V. Kaschula C. H. Maiti P. (2019). Health Benefits of Resveratrol: Evidence from Clinical Studies. Med. Res. Rev. 39, 1851–1891. 10.1002/med.21565 30741437

[B43] Smoliga J. M. Blanchard O. (2014). Enhancing the Delivery of Resveratrol in Humans: If Low Bioavailability Is the Problem, what Is the Solution? Molecules 19, 17154–17172. 10.3390/molecules191117154 25347459 PMC6270951

[B44] Soner B. C. Murat N. Demir O. Guven H. Esen A. Gidener S. (2010). Evaluation of Vascular Smooth Muscle and Corpus Cavernosum on Hypercholesterolemia. Is Resveratrol Promising on Erectile Dysfunction? Int. J. Impot. Res. 22, 227–233. 10.1038/ijir.2010.8 20596084

[B45] Tan C. S. Loh Y. C. Tew W. Y. Yam M. F. (2020). Vasorelaxant Effect of 3,5,4'-Trihydroxy-Trans-Stilbene (Resveratrol) and its Underlying Mechanism. Inflammopharmacology 28, 869–875. 10.1007/s10787-019-00682-6 31925617

[B46] Tsai C. C. Lee M. C. Tey S. L. Liu C. W. Huang S. C. (2017). Mechanism of Resveratrol-Induced Relaxation in the Human Gallbladder. BMC Complement. Altern. Med. 17, 254. 10.1186/s12906-017-1752-x 28482835 PMC5422932

[B47] Tsai C. C. Tey S. L. Lee M. C. Liu C. W. Su Y. T. Huang S. C. (2018). Mechanism of Resveratrol-Induced Relaxation of the guinea Pig Fundus. Phytomedicine 43, 55–59. 10.1016/j.phymed.2018.03.061 29747754

[B48] Van Geldre L. A. Lefebvre R. A. (2004). Interaction of NO and VIP in Gastrointestinal Smooth Muscle Relaxation. Curr. Pharm. Des. 10, 2483–2497. 10.2174/1381612043383890 15320758

[B49] Vesely O. Baldovska S. Kolesarova A. (2021). Enhancing Bioavailability of Nutraceutically Used Resveratrol and Other Stilbenoids. Nutrients 13, 3095. 10.3390/nu13093095 34578972 PMC8470508

[B50] Wang P. Sang S. (2018). Metabolism and Pharmacokinetics of Resveratrol and Pterostilbene. BioFactors 44, 16–25. 10.1002/biof.1410 29315886

[B57] Wang W. Huang H. Hou D. Liu P. Wei H. Fu X. (2010). Mechanosensitivity of STREX-lacking BK_Ca_ Channels in the Colonic Smooth Muscle of the Mouse. Am. J. Physiol. - Gastrointest. Liver Physiol. 299, 1231–1240. 10.1152/ajpgi.00268.2010 20864656

[B51] Wu C.-H. Shieh T.-M. Wang K.-L. Huang T.-C. Hsia S.-M. (2015). Quercetin, a Main Flavonoid in Onion, Inhibits the PGF2α-Induced Uterine Contraction *In Vitro* and *In Vivo* . J. Funct. Foods 19, 495–504. 10.1016/j.jff.2015.09.028

[B58] Xin X. F. Cheng Y. Ren J. Zhang S. Liu P. Zhao H. (2018). The Extracellular Loop of the Auxiliary β1-Subunit is Involved in the Regulation of BK_ca_ Channel Mechanosensitivity. Am. J. Physiol. - Cell Physiol. 315, C485–C493. 10.1152/ajpcell.00037.2018 29924635

[B52] Zhang J. Halm S. T. Halm D. R. (2012). Role of the BK Channel (KCa1.1) during Activation of Electrogenic K+ Secretion in guinea Pig Distal colon. Am. J. Physiol. Gastrointest. Liver Physiol. 303, G1322–G1334. 10.1152/ajpgi.00325.2012 23064759 PMC3532550

[B53] Zhang L. X. Li H. F. Wang L. D. Jin S. Dou X. C. Tian Z. F. (2014). Resveratrol and Genistein Inhibition of Rat Isolated Gastrointestinal Contractions and Related Mechanisms. World J. Gastroenterol. 20, 15335–15342. 10.3748/wjg.v20.i41.15335 25386082 PMC4223267

