# Supplementary material for: Resveratrol Relaxes Human Gastric Smooth Muscles Through High Conductance Calcium-Activated Potassium Channel in a Nitric Oxide-independent Manner
Source: Front Pharmacol. 2022 Jan 25;13:823887. doi: 10.3389/fphar.2022.823887 (PMC8822120; doi:10.3389/fphar.2022.823887)
Supplement: Supplementary file 1 [file Table1.DOCX]

Table S1. The AUC for resveratrol on carbachol-induced contractility of the human ventricle muscles to different antagonist. The values are mean ± SEM of n =10 individual gastric strips from different patients. *p<0.05, **p<0.01, ***p<0.001, ****p<0.001 versus resveratrol alone; ^¥^p<0.05 versus preincubation with IbTX.

| Resveratrol after preincubation with antagonist: | Resveratrol concentration (log mol/L) | | | | | | |
| --- | --- | --- | --- | --- | --- | --- | --- |
|  | **-7** | **-6.5** | **-6** | **-5.5** | **-5** | **-4.5** | **-4** |
| Resveratrol alone | 97.20±0.57 | 96.11±0.71 | 95.86±0.74 | 95.38±1.02 | 93.33±0.93 | 91.50±1.67 | 83.49±2.85 |
| L-NNA | 95.80±1.42 p=0.61 | 94.5±1.42 p=0.67 | 92.56±1.41 p=0.18 | 90.76±1.69* p<0.05 | 89.48±1.87 p=0.15 | 85.00±2.45* p<0.05 | 80.55±3.75 p=0.61 |
| L-NAME | 95.72±0.49 p=0.09 | 93.41±0.86 p=0.051 | 91.87±1.22* p<0.05 | 90.15±1.58* p<0.05 | 87.56±2.47 p=0.06 | 82.95±3.04* p<0.05 | 72.84±3.64* p<0.05 |
| ODQ | 95.83±0.78 p=0.24 | 94.82±0.88 p=0.24 | 94.12±0.99 p=0.14 | 94.35±1.51 p=0.021 | 93.33±2.13 p=0.48 | 88.99±2.75 p=0.34 | 76.40±3.82 p=0.070 |
| TEA | 100.5±0.56*** p<0.001 | 101.6 ±0.83**** p<0.0001 | 101.1±1.11*** p<0.001 | 99.06±1.38* p<0.05 | 95.93±1.87 p=0.47 | 93.67±1.79 p=0.36 | 91.08±2.07* p<0.05 |
| IbTX | 98.59±0.93 p=0.14 | 98.08±1.30 p=0.16 | 98.00±1.51 p=0.18 | 97.99±1.99 p=0.42 | 97.88±1.53* p<0.05 | 97.18±1.40 p=0.14 | 95.60±1.52** p<0.01 |
| ChTX | 98.59±0.93 p=0.14 | 98.05±1.30 p=0.16 | 98.15±1.25 p=0.23 | 97.49±1.99 p=0.61 | 95.12±1.88 p>0.99 | 93.41±1.95 p=0.47 | 89.58±1.98^¥^ p<0.05 |
| Apamin | 94.15±1.15* p<0.05 | 92.62±1.23 p=0.05 | 90.79±1.59* p<0.05 | 89.00±1.71** p<0.01 | 86.71±1.99* p<0.0001 | 81.41±2.40*** p<0.001 | 69.96±3.72* p<0.05 |
| Glibenclamide | 96.69±1.97 p=0.62 | 96.52±1.46 p>0.99 | 93.78±2.06 p=0.32 | 91.98±2.55 p=0.30 | 90.16±3.14 p=0.58 | 86.05±3.41 p=0.10 | 80.59±3.97 p=0.490 |
| 4AP | 96.82±1.96 p=0.34 | 98.67±1.61 p=0.43 | 97.4±1.59 p=0.10 | 93.61±1.80* p<0.05 | 88.91±2.37 p=0.31 | 81.32±3.52** p<0.01 | 75.68±4.13 p=0.09 |
| Tamoxifen | 96.08±1.03 p=0.34 | 93.51±1.23 p=0.16 | 91.90±1.92 p=0.21 | 88.53±2.82**** p<0.0001 | 85.57±3.05 p=0.17 | 80.65±4.34 p=0.16 | 73.17±4.74 p=0.14 |
