# Supplementary material for: Resveratrol Relaxes Human Gastric Smooth Muscles Through High Conductance Calcium-Activated Potassium Channel in a Nitric Oxide-independent Manner
Source: Front Pharmacol. 2022 Jan 25;13:823887. doi: 10.3389/fphar.2022.823887 (PMC8822120; doi:10.3389/fphar.2022.823887)
Supplement: Supplementary file 2 [file Table2.DOCX]

Table S2. The basal tension for resveratrol on carbachol-induced contractility of the human ventricle muscles to different antagonist. The values are mean ± SEM of n =10 individual gastric strips from different patients. *p<0.05, **p<0.01 versus resveratrol alone.

| Resveratrol after preincubation with antagonist: | Resveratrol concentration (log mol/L) | | | | | | |
| --- | --- | --- | --- | --- | --- | --- | --- |
|  | **-7** | **-6.5** | **-6** | **-5.5** | **-5** | **-4.5** | **-4** |
| Resveratrol alone | 97.81±0.94 | 96.70±0.72 | 96.37±1.20 | 96,37±1.20 | 96.72±1.47 | 94.80±1.25 | 88.88±2.74 |
| L-NNA | 97.26±1.57 p=0.50 | 95.80±1.48 p=0.91 | 95.12±2.00 p=0.91 | 95.12±2.00 p=0.91 | 93.93±2.88 p=0.89 | 91.18±3.29 p=0.91 | 85.62±4.13 p=0.97 |
| L-NAME | 95.19±0.82* p<0.05 | 95.03±0.96 p=0.32 | 93.62±2.04 p=.39 | 93.15±2.04 p=0.39 | 92.06±3.00 p=0.22 | 87.69±3.67 p=0.25 | 78.25±4.19 p=0.07 |
| ODQ | 96.25±1.63 p=0.24 | 97.69±1.48 p=0.74 | 99.66±2.30 p=0.28 | 99.66±2.30 p=0.28 | 99.87±2.63 p=0.39 | 96.78±3.45 p=0.48 | 85.42±4.54 p=0.44 |
| TEA | 100.7±0.72** p<0.01 | 101.9 ±1.03** p<0.01 | 100.6±1.11* p<0.05 | 100.6±1.11* p<0.05 | 97.35±1.15 p=0.47 | 95.37±1.01 p=0.43 | 93.12±1.05 p=0.16 |
| IbTX | 98.47±1.75 p=0.46 | 97.87±1.63 p=0.48 | 99.70±1.73 p=0.07 | 99.70±1.73 p=0.07 | 98.19±1.98 p=0.48 | 97.59±1.99 p=0.19 | 96.81±2.05 p=0.06 |
| ChTX | 97.91±1.76 p=0.34 | 96.99±1.17 p>0.99 | 98.94±2.80 p=0.62 | 98.94±2.80 p=0.62 | 97.78±2.97 p=0.84 | 95.88±2.72 p=0.63 | 94.57±2.89 p=0.25 |
| Apamin | 93.97±1.45* p<0.05 | 95.63±1.43 p=0.81 | 92.15±2.00 p=0.25 | 92.15±2.00 p=0.25 | 90.92±2.07* p<0.05 | 86.44±2.56* p<0.05 | 75.40±4.12* p<0.05 |
| Glibenclamide | 97.26±1.94 p=0.36 | 95.35±1.77 p=0.74 | 95.81±3.37 p=0.74 | 95.81±3.37 p=0.74 | 93.86±3.92 p=0.97 | 90.11±4.49 p=0.53 | 83.01±4.23 p=0.31 |
| 4AP | 97.68±2.49 p=0.23 | 99.92±1.91 p=0.07 | 96.36±2.29 p=0.67 | 96.36±2.29 p=0.67 | 94.26±2.22 p=0.34 | 86.40±3.11* p<0.05 | 80.53±3.66 p=0.21 |
| Tamoxifen | 97.74±1.27 p=0.31 | 96.41±1.68 p=0.91 | 93.58±3.22 p=0.58 | 93.58±3.22 p=0.58 | 91.65±3.71 p=0.25 | 88.01±4.64 p=0.28 | 80.51±4.63 p=0.12 |
